# Molecular Aspects of Viral Pathogenesis in Emerging SARS-CoV-2 Variants: Evolving Mechanisms of Infection and Host Response

**DOI:** 10.3390/ijms27020891

**Published:** 2026-01-15

**Authors:** Sofia Teodora Muntean, Andreea-Raluca Cozac-Szoke, Andreea Cătălina Tinca, Irina Bianca Kosovski, Silviu Vultur, Mara Vultur, Ovidiu Simion Cotoi, Anca Ileana Sin

**Affiliations:** 1 Doctoral School of Medicine and Pharmacy, George Emil Palade University of Medicine, Pharmacy, Science, and Technology of Târgu Mures, 540139 Targu Mures, Romania; sofia.harsan@umfst.ro; 2 Department of Pathophysiology, George Emil Palade University of Medicine, Pharmacy, Science, and Technology of Târgu Mures, 540139 Targu Mures, Romania; andreea-catalina.tinca@umfst.ro (A.C.T.);; 3 Pneumology Department, Clinical County Hospital of Mureș, 540142 Targu Mures, Romania; 4 Pathology Department, Clinical County Hospital of Mureș, 540011 Targu Mures, Romania; 5 Clinical Laboratory Department, Clinical County Hospital of Mureș, 540072 Targu Mures, Romania; 6 Plastic, Aesthetic and Reconstructive Microsurgery Department, County Emergency Clinical Hospital of Târgu Mureș, 540136 Targu Mures, Romania; silviu.vultur@yahoo.com (S.V.); nemes.mara.1997@gmail.com (M.V.); 7 Department of Genetics and Cellular and Molecular Biology, George Emil Palade University of Medicine, Pharmacy, Science, and Technology of Târgu Mures, 540139 Targu Mures, Romania

**Keywords:** COVID-19, SARS-CoV-2, viruses, viral pathogenesis, molecular virology, viral entry

## Abstract

Although the SARS-CoV-2 pandemic no longer poses a global emergency, the virus continues to diversify and acquire immunoevasive properties. Understanding the molecular pathways that shape SARS-CoV-2 pathogenesis has become essential. In this paper, we summarize the most recent current evidence on how the spike protein structurally evolves, on changes in key non-structural proteins, such as nsp14, and on host factors, such as TMPRSS2 and neuropilin-1. These changes, together, shape viral entry, replication fidelity and interferon antagonism. Given the emerging Omicron variants of SARS-CoV-2, recent articles in the literature, cryo-EM analyses, and artificial intelligence-assisted mutational modeling were analyzed to infer and contextualize mutation-driven mechanisms. It is through these changes that the virus adapts and evolves, such as optimizing angiotensin-converting enzyme binding, modifying antigenic surfaces, and accumulating mutations that affect CD8^+^ T-cell recognition. Multi-omics data studies further support SARS-CoV-2 pathogenesis through convergent evidence linking viral adaptation to host immune and metabolic reprogramming, as occurs in myocarditis, liver injury, and acute kidney injury. By integrating proteomic, transcriptomic, and structural findings, this work presents how the virus persists and dictates disease severity through interferon antagonism (ORF6, ORF9b, and nsp1), adaptive immune evasion, and metabolic rewiring. All these insights underscore the need for next-generation interventions that provide a multidimensional framework for understanding the evolution of SARS-CoV-2 and guiding future antiviral strategies.

## 1. Introduction

Since the emergence of the virus from the Coronaviridae family, the SARS-CoV-2 coronavirus, and the global COVID-19 pandemic that it triggered have posed unprecedented challenges to the well-being and public health of the entire globe. Following the end of the acute pandemic phase, it has become very clear that this virus is capable of undergoing complex and rapid mutations and using them to exploit host vulnerabilities. Successive variants have highlighted important shifts in viral transmissibility and host–virus interactions, particularly those related to immune evasion and tissue-specific pathogenicity. The SARS-CoV-2 virus exhibits remarkable molecular plasticity and continued discoveries in viral evolution and host responses keep this topic highly relevant, even six years after its emergence. In this context, the integrated analysis of viral mutations and host responses become essential for anticipating evolutionary trends and for developing therapeutic methods capable of responding to future viral pandemics [1]. At the molecular level, SARS-CoV-2 infection is triggered by a complex sequence of interactions between viral proteins and host molecules. The infection process is initiated by the binding of the Spike protein to the angiotensin-converting enzyme 2 receptor and followed by the uptake of the viral protein by cellular proteases such as TMPRSS2, cathepsin L, and furin. In addition, neuropilin-1 is the molecule that acts as a co-receptor and enhances the efficiency of viral entry [2]. All these interactions facilitate membrane fusion and viral entry into target cells, where viral RNA is released and viral genomic replication begins.

Upon initiation of SARS-CoV-2 replication in host cells, the host innate immune system recognizes viral components through pattern recognition receptors (PRRs), such as Toll-like receptors (TLRs), RIG-I, and MDA5, and their activation triggers intracellular signaling cascades. Cellular signaling involves the transcription factor NF-κB and MAPK kinases, leading to the production of type I interferons and the release of proinflammatory cytokines. Dysregulation of these sensing mechanisms, through an excessive inflammatory response, can lead to severe tissue damage, acute respiratory distress syndrome, and multiorgan dysfunction. At the same time, the activation of cyclooxygenase-2, from the arachidonic acid cycle, contributes to the intensification of inflammation and systemic symptoms, thus being a determinant of the clinical severity of the disease [3].

Current research related to the continuous evolution of the SARS-CoV-2 virus, an evolution characterized by the emergence of successive variants with mutations in the Spike protein binding domains, has as central topics the mechanisms of molecular adaptation and immune evasion and provides essential information for understanding the dynamics of infection and the persistence of the virus in the human population. Through all these changes, the SARS-CoV-2 virus can alter the binding affinity to host receptors, can modify the recognition by neutralizing antibodies and can influence adaptive cellular responses.

Our work aims to analyze and synthesize the most recent discoveries regarding the molecular mechanisms by which emerging variants of the SARS-CoV-2 virus influence the processes of infection, replication and the immune response of the host. This review specifically focuses on late Omicron sublineages (BA.2.86/JN.1 and KP descendants) and integrates spike protein evolution with non-spike replication fidelity (nsp14), interferon antagonism, and organ-level multi-omics signatures. We would like to highlight the importance of integrating omics technologies such as genomics, proteomics and transcriptomics, together with structural biological approaches, with which the virus–host interaction has been detailed and potential therapeutic targets have been identified. The multidimensional perspective that we have addressed in this work contributes to the understanding of how viral evolution redefines the molecular landscape of pathogenesis and provides directions for the development of next-generation antiviral strategies.

Unless otherwise specified, experimental evidence in this review refers to findings derived from in vitro, in vivo, or clinical studies. In contrast, results from computational modeling, AI-assisted analyses, and multi-omics datasets are discussed as associative or predictive insights and should not be interpreted as direct causal mechanisms, without functional validation.

While previous reviews have largely focused on individual aspects of SARS-CoV-2 pathogenesis, this review integrates recent structural, multi-omics, and computational data to provide a cross-scale perspective on how late Omicron sublineages reshape viral-host interactions. By linking spike evolution, nonstructural protein function, interferon antagonism, and organ-specific immunometabolic signatures, we aim to highlight emerging unifying mechanisms that may guide therapeutic and vaccine strategies in the future.

## 2. Viral Entry and Receptor Interactions

### 2.1. Spike Protein Evolution

Since the beginning of the pandemic, the spike protein of the SARS-CoV-2 virus has been identified as a distinct mechanism of the pathogenesis of this virus. The transmissibility of the virus is strongly influenced by the changes that occur in the binding sites of the spike protein receptor, and with these mutations, new variants of the virus also appear. Wang, S. et al., in their work published in August 2025, used the Nexclade server, known for identifying mutations of the SARS-CoV-2 virus, and analyzed the main circulating sublines that presented the most important mutations with an impact on the viral behavior: BA.5.2*, XBB.1.5*, XBB.1.16*, XBB.1.22* and XBB.1.9*, and the mutations E748V, H146K, I210T and L455F proved to be particularly relevant in the dynamics of infection and the adaptive capacity of the virus [4]. In this study, the L455F mutation was shown to significantly increase the affinity of the Spike protein for the ACE2 receptor, thereby facilitating efficient entry into host cells.

**Figure 1:**
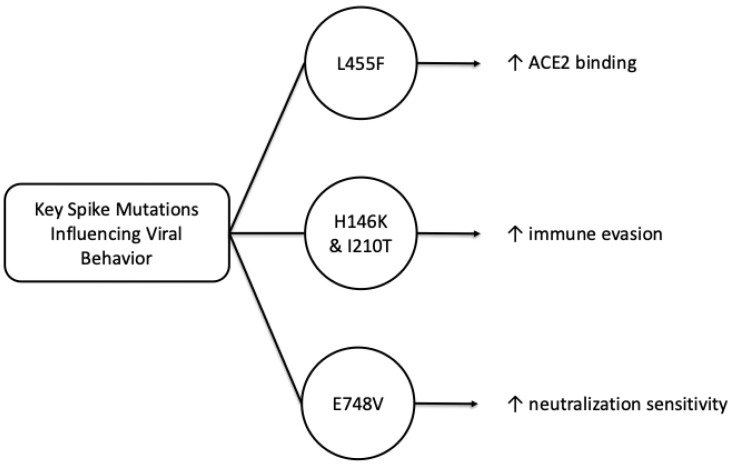
Key Spike protein mutations in recent SARS-CoV-2 Omicron sublineages influencing viral behavior. Schematic summary of selected Spike substitutions discussed in Section 2.1 and their reported functional associations: L455F with increased ACE2 binding, H146K/I210T with increased immune evasion, and E748V with increased neutralization sensitivity.

In parallel, results obtained by the pseudovirus neutralization assay (PVNA) showed that the H146K and I210T mutations increase the immune evasion capacity of the XBB.1.22.1 subline, while the L455F mutation confers a similar effect on transmissibility to the EG.5.1 and EG.5.1.1 sublines. In contrast, the E748V mutation appears to increase the neutralization sensitivity of the XBB.1.5.24 variant, suggesting a trade-off between binding affinity and antibody vulnerability to these SARS-CoV-2 variants [5]. The E748V, I210T and L455F mutations, characteristic of lineages derived from subtypes GF.1, FY.3.1 and HK.3, have been associated with longer epidemic persistence of SARS-CoV-2. The findings in this study highlight the particular importance of continuous monitoring of Spike protein mutations, as they not only influence transmissibility, but also influence the host immune response along with the efficacy of current vaccines. Thus, the integration of multi-omics and structural methods in analyzing and monitoring the evolution of the Spike protein provides a complex insight into the molecular adaptation mechanisms underlying the replicative success and prevalence of new variants of the SARS-CoV-2 virus [6]. Figure 1 provides a simplified overview of three mutation-linked trends discussed here: enhanced ACE2 binding (L455F), immune evasion (H146K/I210T) and increased neutralization sensitivity (E748V).

### 2.2. Structural and Binding Consequences from Cryo-EM/Computational Models

Recently, important advances have been made in cryo-electron microscopy (Cryo-EM) and artificial intelligence-assisted structural modeling, such as AlphaFold, as well as artificial intelligence-based prediction platforms, which have allowed the detailed characterization of molecular changes induced by mutations in the Spike protein of the SARS-CoV-2 virus. With these three-dimensional approaches, it is possible to model and predict how the Omicron subtype-derived KP.3.1.1 variant may adjust its conformation to balance structural stability and ACE2 binding efficiency [7]. In the study, published in July 2025, by Feng Z. et al. [8] on cryo-microscopy found that the deletion of S31 in the N-terminal region led to the emergence of a new glycanization site at residue N30 and changes in the glycan structure at position N61. These changes did not significantly affect the overall architecture of the Spike trimer nor the distribution between the “up” and “down” conformational states of the receptor binding domain (RBD). However, such changes may influence the local mobility of the side chains and potentially alter the exposure of epitopes recognized by neutralizing antibodies, as suggested by structural modeling [9].

Also using Cryo-EM technology, the link between the F456L and Q493E mutations was discovered, which has an epistatic effect on the structure and function of the Spike-hACE2 complex [10]. Surprisingly, they do not act independently, but through an evolutionary process of optimizing molecular interactions, modulate the binding affinity between the RBD and ACE2. The study by Lee et al. [11] found that based on the superposition of Cryo-EM models and AlphaFold predictions, subtle changes in interprotomeric angles and relative distances between the NTD, SD1/SD2, and RBDs were confirmed. This may alter trimer dynamics and potentially influence receptor engagement.

**Figure 2:**
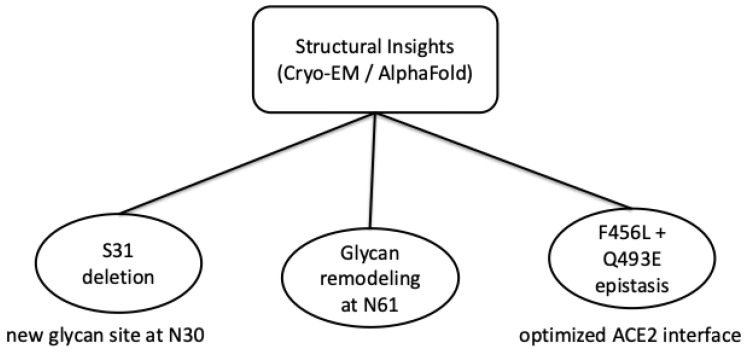
Structural effects of Spike protein mutations of SARS-CoV-2 inferred from cryo-EM and AI-assisted modeling. Schematic overview of key structural features reported for recent Omicron sublineages, including the S31 deletion associated with the emergence of a novel glycosylation site at N30; glycan remodeling at N61; epistatic interactions between F456L and Q493E that optimize the Spike- ACE2 interface. These features are primarily derived from cryo-EM reconstructions and AlphaFold-based structural predictions.

From an evolutionary perspective, the study suggested that these recent mutations, including the reintroduction of residues N30 and Q493E, represent compensatory adaptations that arose under the selective pressure exerted by the host immune response. Such changes in the molecular structure of the Spike protein are proposed to contribute to a balance between structural stability, preservation of binding function and immune evasion, based primarily on structural and computational analyses [7,8,9,10,11]. Figure 2 summarizes how cryo-EM and AlphaFold-based models indicate coordinated Spike adaptations, including glycan remodeling and epistatic mutations that fine-tune ACE2 binding in recent Omicron variants. The integration of high-resolution structural analyses and the use of predictive computational models provides a complex understanding of how successive mutations redraw the conformational landscape of the Spike protein, maintaining infectious competence and adapting the SARS-CoV-2 virus to a dynamic epidemiological context.

Wee J. et al., in their study published in April 2025 [12], focused on detecting viral evolution using deep topological learning assisted by AlphaFold 3, found that this recent computational modeling method can offer promising solutions in anticipating emerging dominant variants of the SARS-CoV-2 virus. It should also be noted that these approaches rely heavily on extensive deep mutational scanning data and accurate three-dimensional models of protein-protein complexes. One of the innovative state-of-the-art strategies, called MT-TopLap (multitask topological Laplacian), which is assisted by AlphaFold 3, integrates machine learning with topological data analysis. The aim is to extract fine geometric and structural features of viral interactions, allowing for the precise estimation of binding free energy changes associated with viral mutations. By validating on four experimental sets of deep mutational scanning of the Spike receptor-angiotensin converting enzyme complex, the AF3-MT-TopLap approach demonstrated notable performance, and maintained a Pearson correlation coefficient of 0.81, even adapting to new variants such as HK.3. Through these methods of integrating structural artificial intelligence with topological analyses, predictive frameworks can be developed that may support rapid response to viral evolution and identifying, in an accelerated manner, mutations with major impact on the transmissibility of the SARS-CoV-2 virus and the immune efficacy of the host. [12]

### 2.3. Role of Co-Factors: TMPRSS2, NRP1, and Potential Alternate Receptors

The entry of SARS-CoV-2 into host cells is a complex process, dependent on a coordinated network of interactions between the viral Spike protein and various host factors. Although the main receptor of the virus, angiotensin-converting enzyme 2 (ACE2), represents the essential anchoring point for the initiation of infection, the actual efficiency of viral entry is largely determined by the presence of cellular cofactors [13]. These cofactors facilitate membrane fusion and expand the cellular spectrum of tropism. Among these, transmembrane serine protease 2 (TMPRSS2) plays a central role in the “activation” of the Spike protein by cleaving it at the S1/S2 and S2′ sites. This proteolytic step is indispensable for the exposure of the fusion peptide and for the efficient fusion of the viral membrane with that of the host cell.

De Carvalho et al. [14] showed in their study that high expression of TMPRSS2 in the upper and lower respiratory epithelium correlates with increased susceptibility to infection, and inhibition of this protease by agents such as camostat mesylate significantly reduces the rate of viral entry. SARS-CoV-2 may utilize alternative pathways of cell entry, involving cathepsin-dependent endocytosis, as demonstrated by infection of tissues with reduced expression of TMPRSS2.

Neuropilin-1 (NRP1), another important determinant of viral infectivity, which is actually a surface glycoprotein involved in cell signaling and angiogenesis, has recently been identified as a coreceptor for the entry of the SARS-CoV-2 virus into the cell. NRP1 first recognizes the polybasic sequence, cleaves it and then exposes it to furin, through the S1 subunit of the Spike protein, thus facilitating viral attachment and internalization. The study by Dette A. et al. [15], focused on the viral entry proteins neuropilin 1 and neuropilin 2, showed that NRP1 is abundantly expressed in endothelial cells, macrophages and mast cells in the lungs, myocardium and nervous tissue, including in cells with low levels of ACE2 and TMPRSS2. The widespread distribution of NRP1 explains, from a molecular point of view, the systemic dissemination of the virus and the multiorgan involvement observed in patients with severe COVID-19. In addition, the detection of viral RNA in cells expressing NRP1, but not ACE2, suggests the existence of alternative mechanisms of viral attachment and entry, in which neuropilins may act independently or in cooperation with other membrane molecules. In this context, other potential surrogate receptors have been proposed, such as AXL (receptor tyrosine kinase), CD147 (Basigin) and cell surface heparan sulfate [16], which may contribute to the initial anchoring of virions and the stabilization of the Spike-receptor complex.

Therefore, the entry of SARS-CoV-2 is not a singular process, but a multifactorial phenomenon, determined by complementary interactions between the Spike protein, host proteases and membrane coreceptors. The molecular redundancy that confers the virus remarkable evolutionary plasticity allows it to colonize diverse tissues and maintain its infectivity even in the presence of genetic variations in the main receptor [17]. Understanding these cofactorial mechanisms opens important directions for the development of combined antiviral strategies that simultaneously target proteases, receptors and alternative entry pathways, thus limiting viral adaptability and the spread of emerging variants.

Building on the molecular determinants of viral entry and host receptor interactions, the next key phase of SARS-CoV-2 pathogenesis involves membrane fusion and the formation of replication complexes that enable efficient viral genome amplification. Beyond receptor engagement and entry cofactors, successful infection requires efficient membrane fusion and the coordinated assembly of replication complexes that sustain viral genome amplification.

## 3. Membrane Fusion and Replication Complexes

The ability of the Spike protein to mediate fusion between the viral and host cell membranes is one of the defining features of the molecular pathogenesis of the SARS-CoV-2 virus and is also a vital process for the initiation of infection. This step depends on a precise sequence of proteolytic events involving the cleavage of furin at the interface of the S1 and S2 subunits of the Spike protein. Other coronaviruses, such as SARS-CoV, do not have the polybasic cleavage site (RRAR) in their structure [18], but it represents a key determinant of the infectivity of the SARS-CoV-2 virus. The activity of the furin protease in the Golgi apparatus of host cells ensures the preactivation of the Spike protein before exposure to the viral surface and thus facilitates a faster and more efficient fusion mechanism upon contact with the ACE2 receptor [19].

A recent study of emerging variants by Silvia-Rios A.A. et al. [20], including variants BA.2.86, JN.1 and KP.3.1.1, revealed optimizations of the furin site by amino acid substitutions adjacent to the RRAR sequence, which may increase enzymatic accessibility and stabilize the prefusion conformation of the Spike protein. Through these modifications, the SARS-CoV-2 virus is suggested to exhibit increased fusion efficiency and an expanded capacity to infect different cell types, from respiratory epithelial cells to endothelial, cardiac and neuronal cells [20]. However, viral tropism and systemic dissemination are inherently multifactorial processes that also depend on ACE2 distribution, the availability of host proteases, entry cofactors such as neuropilin-1, and tissue-specific immune environments. Therefore, even subtle variations in the S1/S2 cleavage region can directly influence the systemic dissemination of the virus and the severity of clinical manifestations. The authors explain that fusion itself is triggered after a second proteolytic event, carried out by TMPRSS2 or endosomal cathepsins, which expose the fusion peptide (FP) from the S2 subunit, which inserts into the host membrane, forming a structural folding complex that brings the two membranes closer together until fusion. Cryo-electron microscopy and molecular dynamics simulations have shown that the transition from the prefusion to the postfusion state involves a major reorganization of the HR1 and HR2 domains and generates a “six-helix bundle” structure, which is characteristic of coronaviruses [18]. Subsequently, the release of the RNA genome into the cytoplasm will trigger the formation of replication-transcription complexes (RTCs), located in membrane compartments derived from the endoplasmic reticulum. These complexes include the nonstructural proteins nsp3, nsp4, and nsp6, which remodel cell membranes to form niches protected by double-enveloped vesicles in which the virus replicates its RNA and synthesizes viral proteins.

The optimization of the furin cleavage site, together with the efficiency with which the membrane reorganizes, reflects a major evolutionary adaptation of the SARS-CoV-2 virus and its variants, which confers a replicative advantage and an increased potential for transmission and infectivity between hosts. By expanding the spectrum of tropism and accelerating the infectious cycle, these changes contribute to increased pathogenicity and the ability of the virus to persist and evolve in the human population, even in the presence of immune or therapeutic pressures.

### Mutations in SARS-CoV-2 Nonstructural Proteins: Focus on nsp14 (ExoN/N7-MTase)

Coronaviruses synthesize a 5′ RNA cap in the cytoplasm to mimic host mRNAs, thereby ensuring efficient viral replication while avoiding immune sensors in the cell. In SARS-CoV-2 pathogenesis, this process relies largely on nonstructural protein 14 (nsp14), a dual-function enzyme that integrates a 3′-5′ exoribonuclease (ExoN). This exoribonuclease is responsible for proofreading with N7-methyltransferase, which will catalyze the methylation of guanine-N7 during RNA cap formation. Structural and mutational analyses have indicated that these two domains operate in a tightly interconnected architecture and may allow coordinated control of replication fidelity and cap formation. Disruption of either enzymatic activity results in profound reduction in viral viability, thus underscoring the essential role of nsp14 in the SARS-CoV-2 virus life cycle. Beyond its direct involvement in RNA synthesis, nsp14 is capable of reprogramming the host transcriptome, suggesting necessary pathogenic functions that extend beyond its canonical roles in RNA proofreading and capping. Because these activities depend on highly coordinated structural domains, even subtle mutations within nsp14 can disproportionately affect viral replication fidelity, immune evasion, and host cell reprogramming [21].

From an evolutionary perspective, nsp14 is a highly conserved protein, but mutations that occur in functional regions or at the interface with its cofactors can have disproportionate effects. In the ExoN domain, substitutions that affect the catalytic DEDDH motif or the coordination of divalent ions, such as the divalent magnesium ion, can diminish or eliminate proofreading nucleolysis, increasing the replication error rate. The result is increased genetic plasticity, which can accelerate adaptation to selective pressures (immune or therapeutic), but also the risk of error catastrophe if fidelity falls below a critical threshold. In addition, the zinc fingers (ZnF1/ZnF2) of ExoN have structural and substrate recognition roles: mutations in these motifs can destabilize the nsp10-nsp14 assembly or disconnect the RNA from the active site, affecting proofreading and viral viability [22].

The nsp10-nsp14 interface presents several highly sensitive mutations, as they can allosterically reduce ExoN activity, which translates into an increased mutation frequency and an altered response to nucleotide analogs (NA). Similarly, variations in the hinge separating the ExoN and MTase domains disrupt the functional coupling between proofreading and capping, and affect cap(0)-mediated fidelity and immune evasion. In the N7-MTase domain, residues involved in SAM (methyl donor) binding and in the GTP pocket of the cap are critical. Mutations that reduce SAM affinity or reorganize the active microenvironment decrease the efficiency of N7-methylation and generate transcripts with incomplete/aberrant caps. These transcripts are more susceptible to degradation and more visible to PRRs, thus presenting potential consequences on pathogenicity and viral load dynamics. In contrast, stabilizing mutations in N7-MTase could favor more efficient binding, contributing to the long-term viral persistence of SARS-CoV-2 in certain tissue compartments [23].

The interaction between nsp14 and nucleotide analogs used as antiviral proteins is an issue with essential therapeutic implications. Because ExoN can excise certain analogues incorporated at the 3’ end (e.g., a portion of remdesivir-monophosphate-containing transcripts), mutations that reduce proofreading activity may sensitize the virus to such agents, but this comes at the cost of increased genetic instability. On the other hand, analogues that act through lethal mutagenesis (e.g., molnupiravir-like models) can partially bypass ExoN proofreading; in these contexts, mutational deviation of nsp14 could influence both the efficacy and the resistance profile of combination therapy (NA + nsp14/nsp10 inhibitors).

Table 1 describes the functional domains of SARS-CoV-2 nsp14, key mutations and their pathogenic mechanism.

Finally, nsp14 constitutes an attractive target for drugs precisely because of its functional duality and the lack of direct homologs in the human cell. The mutations mapped in the SAM pocket, the RNA channel, the DEDDH motif, the metal chelation sites and the nsp10-nsp14 interface provide structural landmarks for the design of inhibitors: (i) chelators or antagonists that deregulate the metal chemistry in ExoN; (ii) ligands that habituate the catalytic pocket and block RNA access; (iii) PPI disruption molecules at the interface with nsp10; (iv) SAM pocket competitors for N7-MTase, ideally uncharged and selective to overcome the permeability and selectivity limits observed in classical SAM analogs. In this framework, functional mapping of mutations in nsp14 and its partners (nsp10, nsp12, nsp16) becomes essential for the rational optimization of new classes of broad-spectrum antivirals against coronaviruses [24].

## 4. Insights from Omics Technologies

**Figure 3:**
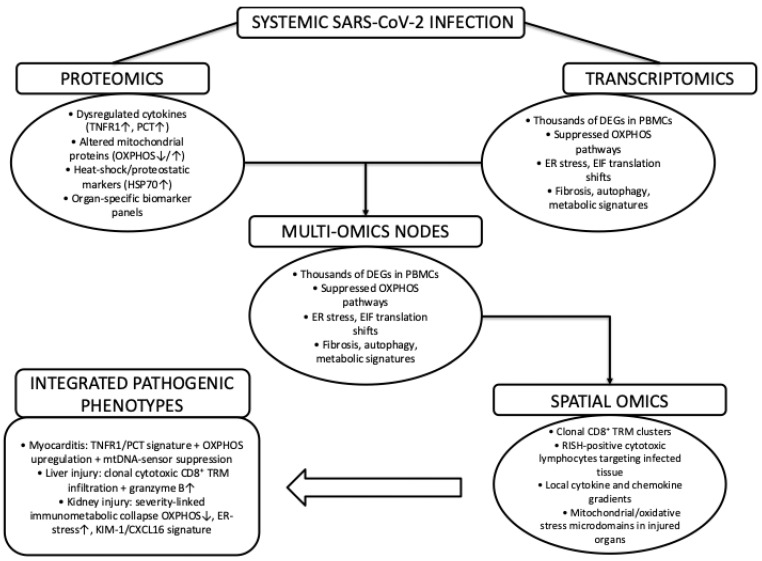
Multi-omics integration of systemic SARS-CoV-2 induced host responses. Schematic representation of how proteomic, transcriptomic, and spatial-omics datasets converge to define systemic and organ-specific pathogenic phenotypes in SARS-CoV-2 infection. Proteomic analyses highlight dysregulated inflammatory mediators, mitochondrial dysfunction, and proteostatic stress, while transcriptomic profiling reveals large-scale transcriptional reprogramming. These include suppression of oxidative phosphorylation pathways and activation of endoplasmic reticulum stress responses. Integration of these datasets identifies shared multi-omics nodes that are further resolved by spatial-omics approaches. Such approaches are linking molecular alterations to tissue-localized immune cell infiltration, metabolic stress microdomains, and distinct clinical phenotypes such as myocarditis, liver injury, and acute kidney injury. The symbol ‘↓’ denotes a decrease, ‘↑’ denotes an increase, and ‘+’ indicates in combination with.

The next natural step would be to ask how these previously described processes are reflected at the systems level in human tissues, having outlined the molecular mechanisms that govern SARS-CoV-2 virus entry, replication, and immune escape. To translate molecular mechanisms into tissue-level pathology, multi-omics approaches provide an essential systems-level perspective. As summarized in Figure 3, proteomic and transcriptomic analyses converge on shared molecular nodes characterized by inflammatory signaling, mitochondrial dysfunction, and proteostatic stress, which are further contextualized by spatial-omics data that resolve organ-specific immune infiltration and metabolic microenvironments. Together, these integrative datasets link systemic SARS-CoV-2 infection to distinct pathogenic phenotypes across the heart, liver, and kidney.

### 4.1. Proteomics and Multi-Omics in COVID-19 Pathogenesis

Proteomics has emerged as a central tool in deciphering the molecular underpinnings of SARS-CoV-2 pathogenesis and is providing vital insights into its pathogenesis that transcend genomic and transcriptomic profiling. By mapping the full repertoire of viral and host proteins, proteomic analyses reveal how SARS-CoV-2 can restructure the cellular machinery to sustain its replication and evade innate immunity. Mass spectrometry has identified extensive remodeling of inflammatory, metabolic, and stress response pathways, including dysregulated cytokine networks, mitochondrial dysfunction, and proteostatic imbalances that collectively contribute to hyperinflammation and multiorgan injury [25].

At the viral level, proteomics defines post-translational modifications of structural and nonstructural proteins that shape infectivity, replication fidelity, and immune escape [25]. Importantly, integrating proteomic signatures with transcriptomic and interactomic datasets has uncovered key molecular nodes, such as NF-κB activation, interferon suppression, and host–virus protein–protein interactions, that underpin disease severity and long term sequelae. Thus, proteomics provides indispensable mechanistic insight into SARS-CoV-2 biology and offers a foundation for the discovery of biomarkers and therapeutic targets.

### 4.2. Cardiac Proteo-Transcriptomic Signatures: SARS-CoV-2 Myocarditis

Early proteomic signatures in SARS-CoV-2-associated myocarditis, as shown in a study published in early 2025 conducted by Pollett S. et al. [26]. Proteomic profiling in the early postinfectious phase of COVID-19 reveals a statistically robust molecular signature in patients who develop SARS-CoV-2-associated myocarditis. Compared with matched SARS-CoV-2-positive controls without myocardial involvement, myocarditis cases showed a significant elevation of TNFR1 (≈0.33 log_10_ pg/mL, *p* = 0.032) and procalcitonin (≈0.59 log_10_ pg/mL, *p* = 0.02), whereas the remaining 18 tested biomarkers showed no meaningful differences emphasizing the selectivity of this proteomic pattern.

It appears that increased TNF signaling and systemic inflammatory activation are key factors involved in the pathogenesis of SARS-CoV-2 myocardial injury. Increased TNFR1 activity mechanistically enhances oxidative stress, endothelial dysfunction, and necroinflammatory lesions and thus promotes the transition from transient inflammatory injury to chronic, sustained myocardial injury. Profound mitochondrial reprogramming, marked by upregulated OXPHOS programs and concomitant suppression of mtDNA-dependent innate immune pathways, data obtained by integrating proteomic and transcriptomic data represent a combination that supports low-grade inflammation while impairing the efficient clearance of viral antigens or DAMPs.

Together, these multi-omics findings suggest the presence of a dominant metabolic cardioinflammatory signature associated with COVID-19-associated myocarditis, in which the interplay between tumor necrosis factor-induced inflammation and mitochondrial dysfunction shapes disease progression toward the long-COVID syndrome. Thus, by identifying TNFR1 and PCT as early biomarkers, they offer promising avenues for improved risk stratification and severity of long-term effects of SARS-CoV-2. From the same perspective, therapeutic strategies targeting TNF signaling, mitochondrial homeostasis, and restoration of mtDNA-mediated antiviral sensing can be designed.

### 4.3. Spatial Multi-Omics in Liver Injury (SVALI)

Spatial proteomics and transcriptomics have also been used to elucidate the immunopathology of SARS-CoV-2-associated liver injury (SVALI). Uzun S. et al. [27] reported a remarkable signature of cytotoxic T cells, dominated by CD8^+^ clonal expansions with a clear statistical and spatial structure. In explanted liver tissue, CD8^+^ lymphocytes constituted ≈17.1% of the total cells, far exceeding CD4^+^ (≈8.7%) and B cells (≈0.5%), while TCRβ-CDR3 sequencing identified 508 distinct T cell clones, including nine hyperexpanded clones (>1% each), with the most dominant one comprising ≈ 14.5% of the entire TCR repertoire. A total of 59 identical clones maintained clonal persistence between biopsy and explant and, surprisingly, three major clones displayed CDR3 motifs similar to Spike-specific responses, suggesting careful selection driven by antigen or by molecular mimicry. Spatial transcriptomics and RISH mapping confirmed that RISH^+^ cells were CD8^+^ positive and preferentially localized to immune-exposed microenvironments such as the portal zone (≈34.3%), portal interface (≈20.9%) and lobular interface (≈12.3%) of the liver. The highest density of such cells was observed at the portal interface, positioning these clones directly adjacent to hepatocytes. Functionally, this population displayed an enhanced cytotoxic profile: ≈21.8% of CD8^+^ RISH^+^ cells were Granzyme B^+^, significantly exceeding CD8^+^ RISH^−^ cells (≈13.2%), with maximal cytotoxicity in extrasinusoidal regions (≈27.5%) and at the lobular interface (≈25.8%). TRM-associated markers were prominently expressed in CXCR6 (≈34.7%), CD69 (≈23.1%), and KLRB1/CD161 (≈47.4%), indicating a tissue-resident effector phenotype rather than an innate MAIT signature.

The convergence of clonal hyperexpansion, spatial clustering at hepatocyte-rich interfaces, and enhanced granzyme-mediated cytotoxicity provides an important pathophysiological basis for hepatocellular injury associated with SARS-CoV-2 infection. From a clinical and practical perspective, the quantitative burden and distribution of CD8^+^ TRM clones may serve as prognostic biomarkers and therapeutic targets in tumor necrosis factor-induced inflammation and mitochondrial homeostasis. Future validation in independent cohorts of liver injury will determine whether these multi-omics signatures reliably stratify risk and disease trajectory in SVALI.

### 4.4. Immunometabolic Signatures in COVID-19-Associated AKI

Multi-omics analysis of COVID-19-induced acute kidney injury (AKI), described by Jayaraman P. et al. [28], provides a comprehensive view of how the kidney responds to SARS-CoV-2 infection, depending on its severity. The study, conducted in hospitalized patients classified according to the degree of renal damage, revealed a clear severity-dependent immunometabolic change that separates mild AKI from the profound dysfunction observed in KDIGO stages 2 and 3. Bulk RNA sequencing of PBMCs, carefully adjusted for clinical variables and differences in immune cell composition, showed that severe AKI is associated with large-scale changes in gene expression. The most pronounced change is the global decrease in mitochondrial transcripts and disruption of OXPHOS pathways, clear indications of a systemic energetic collapse in circulating immune cells.

In parallel, activation of endoplasmic reticulum stress responses, dysregulation of EIF-dependent protein translation modules, enhanced chemokine and cytokine signaling, and alterations in cell adhesion programs were observed [29]. Together, these changes describe a pronounced imbalance between inflammatory and proteostasis-maintaining processes. At the same time, the involvement of important molecular nodes, such as TFAM, in signaling pathways associated with EGFR and the circadian component PER1 was highlighted. In addition, microRNA analysis identified miR-492 as a possible specific regulator of severe forms of COVID-19 disease [30].

These transcriptomic signatures were validated using proteomic technology. Thus, the pathogenesis of SARS-CoV-2 virus, based on systemic inflammation, mitochondrial stress and acid-base imbalance, was confirmed by the presence of HAVCR1/KIM-1, CXCL16, IL17RC, ITGB2, heat shock proteins HSPA1A/HSP70 and DNAJB12/HSP40, as well as the metabolic enzymes CA3 and CA4. When compared with the profiles of sepsis-associated acute kidney injury, both this and that related to COVID-19 disease, showed common activations of apoptotic pathways, endoplasmic reticulum stress and TNF/NF-κB signaling. However, the renal injury associated with COVID-19 showed a much more pronounced collapse of OXPHOS regulation, suggesting that bioenergetic insufficiency is a specific molecular feature of SARS-CoV-2-induced renal injury [31].

Overall, these results suggest that PBMC-derived biomarkers, such as KIM-1, CXCL16, and miR-492, may become useful tools for early risk stratification of renal injury associated with SARS-CoV-2 infection, pending validation in independent and longitudinal cohorts. These represent therapeutic avenues worth exploring, such as interventions that stabilize mitochondrial function, reduce endoplasmic reticulum stress, and restore immunometabolic balance. These strategies may limit progression to severe forms of post-viral acute renal failure and prevent chronic renal dysfunction associated with long-COVID syndrome.

## 5. Immune Evasion and Host–Virus Molecular Interplay

The omics data highlighted in the previous section converge on a common theme: SARS-CoV-2 pathogenesis is tightly linked to how the virus modulates host immune sensing and cellular signaling networks. In this section, we move from organ level signatures back to the mechanistic interface between virus and host, focusing on how SARS-CoV-2 evades interferon responses, escapes adaptive immunity, and rewires intracellular signaling and metabolism.

### 5.1. Interferon Antagonism

As defined by Li D. and Wu M., pattern recognition receptors (PRRs) are a central component of the innate immune system, acting as molecular sentinels that detect invading pathogens and also recognize conserved viral structures, known as pathogen associated molecular patterns (PAMPs), including single stranded and double stranded RNA, viral proteins, or host derived danger signals released during cell injury. Once activated, PRRs initiate signaling cascades that converge on transcription factors such as IRF3/7 and NF-κB, triggering the production of type I and III interferons and proinflammatory cytokines [32]. An overview of SARS-CoV-2 proteins involved in antagonizing PRR sensing and interferon signaling pathways is provided in Table 2.

Several families of PRRs participate in the recognition of SARS-CoV-2: Toll-like receptors (TLRs) located on the cell surface or in endosomes (particularly TLR2, TLR3, TLR4, TLR7, TLR8, and TLR9); RIG-I-like receptors (RLRs), such as RIG-I and MDA5, which detect cytosolic viral RNA; and cytosolic DNA sensors, including cGAS and AIM2, which can respond to virus-induced cellular damage. All of these receptors have a role in the antiviral defense response, but their excessive and dysregulated activation contributes to hyperinflammation, cytokine storm, and a severe form of COVID-19 disease. Furthermore, genetic variations in PRRs, such as TLR3 polymorphisms or X-linked TLR7 deficiency, have been linked to life-threatening diseases, highlighting their role in determining individual susceptibility. Because SARS-CoV-2 has evolved strategies to evade detection by PRRs, understanding PRR signaling is essential for identifying therapeutic targets and designing immunomodulatory approaches for both acute and chronic COVID-19 [33].

SARS-CoV-2 utilizes multiple viral proteins to attenuate pattern recognition receptor (PRR) detection and block interferon (IFN) signaling, allowing the virus to replicate with minimal antiviral pressure. One of the most potent antagonists that exhibits such a mechanism, ORF6, anchors to the nuclear pore complex, where it binds to the Nup98-Rae1 complex and blocks the entry of phosphorylated STAT1 and IRF3 proteins into the nucleus. By preventing these transcription factors from reaching the nucleus, ORF6 effectively uncouples IFN-stimulated gene (ISG) induction from upstream detection. The most recent Omicron variants of SARS-CoV-2 (e.g., BA.4/BA.5) exhibit increased expression of ORF6, which correlates with reduced STAT1 phosphorylation and impaired IFN-I/III signaling in epithelial cells [34].

Another SARS-CoV-2 accessory protein, ORF9b, plays a dual role in shaping innate immunity. In the first phase, it suppresses antiviral interferon signaling and then promotes proinflammatory activity. Beyond the well-described inhibition of the RIG-I/MDA5-MAVS pathways, E. Zodda et al. [35] showed that ORF9b can directly prime and activate the NLRP3 inflammasome, a multiprotein complex responsible for the maturation of IL-1β and IL-18. As a localization, ORF9b is placed inside the mitochondria and disrupts their homeostasis by interacting with TOM70, altering mitochondrial dynamics and promoting the release of mitochondrial reactive oxygen species (mtROS), a potent NLRP3 activator. At the same time, the accessory protein ORF9b can suppress interferon responses and remove inhibitory constraints on inflammasome signaling, thus creating a cellular environment in which NLRP3 activation occurs more robustly. ORF9b interferes with cytosolic viral RNA sensing pathways by directly targeting RIG-I, MDA5, MAVS, TRIF, STING, and TBK1, thereby blocking IRF3 activation and suppressing interferon production at multiple checkpoints. In parallel, the nsp1 protein enhances immune evasion by binding to the 40S ribosomal subunit, obstructing the mRNA entry channel, and inducing selective degradation of host transcripts. This “host shutdown” mechanism limits the translation of antiviral factors, further weakening the interferon response. Together, all of these coordinated strategies are thought to allow recent SARS-CoV-2 variants to more effectively suppress MDA5/RIG-I activation and downstream ISG expression, thereby enhancing viral replication and transmissibility, although the relative contribution of each antagonist can differ by variant and cellular context.

### 5.2. Evading Adaptive Immunity

The accumulation of mutations in the receptor binding domain (RBD) and the N-terminal domain (NTD) of the spike glycoprotein progressively diminishes the binding efficiency of neutralizing antibodies acquired from previous infection or vaccination. Variants BA.2.86 and its descendant JN.1 have managed to adapt to circumvent existing humoral immunity. Findings by Kim T.-H. et al. [36] indicate that repeated exposure to Omicron antigens, either by infection or by booster immunization, can gradually attenuate the strong immune imprint left by ancestral strains. This repeated stimulation promotes a much broader and more flexible repertoire of neutralizing antibodies that can be detected in both plasma and mucosal surfaces, highlighting an essential consideration for future updates of vaccine antigens.

Although T-cell immunity is generally more tolerant of spike variability than antibody responses, they (Kim T.-H. et al.) have identified several CD8^+^ T-cell epitope “hotspots” in the spike and nucleocapsid proteins where mutations associated with JN.1 can impair HLA class I presentation. One such example is the Q229K change in the N protein from HLA-A*02:01-restricted epitopes that has the potential to diminish T-cell recognition, suggesting that selective pressure is also beginning to act on cytotoxic epitopes. It is important to note, however, that many T-cell epitopes, particularly those located in the conserved S2 regions and in nonstructural proteins, remain functionally intact. These stable regions continue to support durable cellular immunity and offer promising targets for the development of next-generation pan-variant vaccines that incorporate highly conserved T-cell epitopes [37].

### 5.3. Host Cell Signaling and Metabolic Reprogramming

Tian J. et al. [38] have documented that the extensive mutational load in the descendants of Omicron variants, particularly BA.2.86 and its rapidly expanding sublineage JN.1, has consequences that extend far beyond classical antibody evasion. These mutations have the capacity to remodel intracellular signaling circuits and disrupt host metabolic homeostasis. The BA.2.86 variant carries more than 30 additional spike protein substitutions and over 60 mutations in the viral proteome, many of which map to or flank CD8^+^ T-cell epitope “hotspots”. These changes allow immune evasion, not only from neutralizing antibodies but also from HLA class I-restricted cytotoxic surveillance. This reduces T-cell-mediated clearance and prolongs intracellular viral residence and thus viral lifespan. This results in amplified signaling disturbances characterized by chronic NF-κB activation, attenuated interferon responses, and hyperactivation of MAPK and JAK/STAT pathways. This defines an inflammatory, but antivirally ineffective state.

The mutations of the JN.1 variant, namely L455S and R3821K, are those that can further disrupt mitochondrial antiviral platforms (MAVS) and homeostasis of the endoplasmic reticulum-Golgi complex [39]. They may contribute to metabolic shifts in the sense of increased glycolytic flux, reduced oxidative phosphorylation, and increased reticular oxidative stress. These immunometabolic changes are proposed to support and promote high-rate viral protein synthesis. But on the other hand, they are affecting antigen processing, autophagic degradation, and detection by the host’s innate immune system [38].

As a host response to SARS-CoV-2 infection, a remodeling of the host’s transcriptomic and proteostatic landscapes occurs. These remodelings act through a combination of nsp1-mediated translational arrest, blockade of nuclear transport by ORF6, and broad modulation of the ER/unfolded protein stress response pathways and JAK/STAT junctions. Accessory proteins, including ORF3c, localize to mitochondria and promote a shift from glycolysis to fatty acid oxidation and OXPHOS. Thereby increasing the production of reactive oxygen species, while autophagic flux is inhibited by impaired lysosomal acidification. Chen et al. [40] have shown in their work that disruption of cGAS-STING signaling and the eIF2/mTOR pathways further entrench a persistent state of inflammation, coupled with metabolic remodeling. They showed that reprogramming of amino acid, cholesterol, and fatty acid metabolism is a defining feature of SARS-CoV-2 pathogenesis, particularly of the recent Omicron variants, and identified actionable nodes for host-directed therapy.

Together, the synergy between interferon antagonism (ORF6, ORF9b, nsp1), adaptive immune evasion mediated by RBD/NTD mutations, and disruption of T-cell epitopes and profound immunometabolic rewiring creates a pathogenic landscape. In this landscape, late Omicron sublineages exhibit stronger suppression of MDA5/RIG-I detection and increased antigenic plasticity. These features underscore the urgency of developing next-generation vaccines and therapies that target the evolving virus–host interface.

## 6. Therapeutic and Vaccine Development Perspectives

Given the molecular mechanisms discussed above, an obvious translational question is how vaccination strategies might keep pace with the constant evolution of the SARS-CoV-2 virus. The continued diversification of Omicron lineages highlights the limits of relying exclusively on variant-compatible neutralizing antibodies and supports a broader approach that combines updated antigen design with immune durability. In this context, next-generation vaccines are increasingly being viewed in terms of scale and site-specific protection, including multivalent formulations, incorporation of more conserved targets, and routes of administration that enhance mucosal immunity [41]. This section therefore summarizes the current vaccine landscape and outlines emerging directions aimed at improving cross-variant protection, reducing breakthrough infections, and maintaining clinical effectiveness in high-risk populations.

The ongoing evolution of the SARS-CoV-2 virus poses significant challenges for the development of antiviral vaccines when targeting rapidly evolving viral proteins. Strategies focused on spike proteins may lose efficacy as new variants accumulate mutations in immunodominant regions. Therefore, complementary approaches that focus on conserved viral elements, including CD8^+^ and CD4^+^ T-cell epitopes located in the S2 domain and nonstructural proteins, are increasingly relevant for the design of pan-variant vaccines [42].

In parallel, host-directed therapeutic strategies offer a means to reduce disease severity while minimizing selective pressure on viral genomes. Multi-omics analyses have helped identify convergent host pathways, including mitochondrial dysfunction, endoplasmic reticulum stress, and dysregulated inflammatory signaling, as central drivers of tissue injury across organs. Targeting these conserved host responses may provide broad therapeutic benefits across variants [43].

The spread of the SARS-CoV-2 virus over the years has shown that rapid antigenic drift, particularly within the Spike protein, can progressively erode the protection of neutralizing antibodies and may require repeated vaccine updates. This evolutionary model supports a shift from purely variant-compatible boosters to next-generation vaccination strategies designed to be scalable and sustainable. These include multivalent formulations, targeting of conserved epitopes (e.g., S2 and non-Spike antigens such as the nucleocapsid), and platforms that better support cellular immunity alongside neutralizing antibodies. In addition, alternative routes of administration may significantly improve real-world efficacy: intranasal or inhaled vaccination. Such methods have the potential to enhance mucosal immunity at the primary site of infection by inducing secretory IgA and tissue-resident memory T cells, thereby reducing transmission and intermittent infections more effectively than purely systemic approaches [44].

From a translational perspective, these vaccine concepts are complemented by host-directed interventions aimed at limiting immunopathology to vulnerable groups, in whom vaccine responses may be suboptimal. Together, the integration of continuous genomic surveillance with flexible antigen design, improved delivery platforms, and immunologically informed targeting of conserved viral regions represents a rational path toward sustained control of COVID-19 disease spread and preparation for future waves of infection.

Large-scale immunoprofiling analyses indicate that repeated exposure to antigenically divergent Omicron sublineages remodels both the humoral and cellular immune landscape, favoring viral spread at the expense of peak neutralizing titers [42]. Rather than inducing narrowly focused antibody responses, updated vaccine formulations appear to promote cross-reactive memory B-cell pools and sustain polyfunctional T-cell responses that are less sensitive to spike mutational drift. Importantly, these immune features correlate more strongly with protection from severe disease than with sterilizing immunity, emphasizing that long-term vaccine effectiveness relies on immune quality rather than antibody magnitude alone [45]. These findings reinforce the rationale for vaccine strategies that prioritize conserved antigenic regions, immune durability, and functional cellular responses, particularly in populations with repeated antigen exposure through infection or boosting. Together, such data provide a mechanistic foundation for the transition toward next-generation vaccines designed to maintain clinical protection despite ongoing viral diversification.

Finally, the integration of real-time genomic surveillance with artificial intelligence-assisted structural modeling and functional validation holds promise for predictive virology, enabling early identification of high-risk variants and accelerating the development of next-generation antiviral interventions.

## 7. Conclusions

The SARS-CoV-2 virus, from an evolutionary perspective, continues to reshape the landscape of viral pathogenesis by altering host receptor interactions, modulating innate and adaptive immunity, and reprogramming host cellular metabolism. Recent variants of the Omicron lineage, including subtypes BA.2.86 and JN.1, demonstrate that this virus adapts through coordinated changes in the Spike protein, the nonstructural replicative machinery, and accessory immunomodulatory proteins. These structural changes enhance viral entry, replication fidelity, interferon antagonism, and antigenic plasticity. Converging patterns of mitochondrial dysfunction, reticulum stress activation, and OXPHOS collapse are revealed using multi-omic datasets, including proteomics, transcriptomics, and spatial omics. Tissue damage caused by SARS-CoV-2 infection, mediated by cytotoxic T cells in the main affected organs, demonstrates the capacity to remodel host systems, beyond the classical respiratory tropism.

With the constant diversification of this virus and its variants, a comprehensive understanding of virus–host molecular interactions is essential to anticipate pathogenic changes and guide therapeutic innovations. The integration of structural biology, artificial intelligence-based mutational prediction, and high-resolution omics now provides a modern multidimensional framework for identifying viral vulnerabilities and dysregulated host pathways. Such insights are and will be crucial for the design of next-generation vaccines, for the development of human antiviral therapies, and for improving the early detection of severe disease phenotypes. Continued global surveillance and interdisciplinary research remain indispensable to maintain preparedness against future evolutionary trajectories of SARS-CoV-2 and also in preparing for other possible future pandemics.

The authors express gratitude to the researchers whose structural, computational, and multi-omic studies continue to expand our understanding of SARS-CoV-2 biology. We acknowledge the scientific community for rapid data sharing, which has enabled real time tracking of viral evolution and facilitated the integration of genomics, proteomics, and computational modeling in this manuscript. No external funding was used for the preparation of this review.

Looking forward, several key research frontiers will be critical for advancing long-term control of SARS-CoV-2 infections and for improving preparedness against future viral threats. The integration of artificial intelligence and machine learning into predictive virology is emerging as a powerful approach for early identification of high-risk variants, enabling the assessment of mutational impacts on transmissibility, immune escape, and pathogenic potential before widespread circulation occurs. In parallel, the expanding application of multi-omics technologies offers an unprecedented opportunity to dissect the molecular basis of Long COVID. These technologies will help in linking persistent clinical symptoms to sustained immunometabolic dysregulation, mitochondrial dysfunction, and tissue-specific inflammatory programs. Finally, these insights should inform the rational design of next-generation therapeutics, including broad-spectrum antivirals and vaccines that target conserved viral structures and stable virus–host interaction interfaces. By focusing on evolutionary constraints rather than rapidly mutating epitopes, such strategies hold promise for achieving durable protection and reducing vulnerability to future waves of viral evolution.

## Figures and Tables

**Table 1:** Functional domains of SARS-CoV-2 nsp14, key mutations and pathogenic or therapeutic implications. Data summarized from structural, biochemical, and functional studies of nsp14 [21,22,23,24].

nsp14 Domain/Region	Key Functions	Impact of Mutations	Pathogenic Consequences
ExoN domain	Proofreading during RNA replication, ensures high-fidelity genome synthesis	Mutations in DEDDH catalytic motif reduce or abolish nuclease activity Disrupted Mg^2+^ coordination impairs proofreading Alterations in ZnF1/ZnF2 destabilize nsp10–nsp14 assembly	Increased mutation rate and viral genetic plasticity Potential “error catastrophe” Enhanced immune evasion due to rapid adaptation
N7-methyltransferase domain	Methylation of guanine-N7 during 5′ RNA capping and shielding viral RNA from PRRs	Mutations in SAM-binding pocket reduce methyl donor affinity Altered GTP-binding site decreases cap efficiency Hinge-region mutations disrupt coupling to ExoN	Production of incomplete/distorted RNA caps Increased RNA detection by MDA5/RIG-I Reduced replicative fitness
nsp10–nsp14 interface	Allosteric activation of ExoN that stabilizes catalytic geometry and RNA-binding channel	Interface mutations weaken nsp10 binding Reduced allosteric stimulation of ExoN	Higher replication error rate Altered response to nucleotide analogs
Replication–transcription complex interactions	Coordinated RNA replication and capping Spatial organization within double-membrane vesicles	Mutations altering domain flexibility disrupt synchronization of replication and capping	Lower replication efficiency Increased exposure of viral RNA to immune sensors
General evolutionary landscape	nsp14 overall is highly conserved Mutations accumulate at constrained functional sites	Large phenotypic shifts due to interlinked domain architecture	Modulates virus evolution, transmissibility, and immune escape

**Table 2:** SARS-CoV-2 proteins reported to antagonize PRR sensing and interferon signaling pathways. Mechanisms and targets are summarized from published experimental studies and reviews [32,33,34,35], with nsp14-specific functions supported by structural and biochemical studies [21,22,23,24]. The symbol ‘↓’ denotes a decrease, whereas ‘↑’ denotes an increase.

Viral Protein	Host Target	Mechanism of Action	Functional Consequence
ORF6	Nup98-Rae1 nuclear pore complex	Blocks nuclear import of pSTAT1 and IRF3	↓ ISG transcription; strong suppression of IFN-I/III
ORF9b	RIG-I, MDA5, MAVS, TOM70, TBK1, STING	Disrupts mitochondrial signaling; inhibits antiviral kinase activation; primes NLRP3 inflammasome	↓ IFN induction; ↑ IL-1β/IL-18 activation; ↑ inflammation
nsp1	40S ribosomal subunit	Occludes mRNA entry channel; degrades host transcripts (“host shutoff”)	↓ translation of antiviral proteins; global suppression of innate immunity
nsp14 (ExoN)	Viral RNA proofreading machinery	Removes incorporated nucleotides, preventing error sensing	↓ exposure of viral RNA to PRRs; ↑ immune evasion
ORF3b/ORF7a	IRF3 pathway components	Blocks IRF3 phosphorylation and nuclear translocation	↓ IFN-I expression
M protein	TBK1	Prevents TBK1 activation	↓ IRF3 activation; ↓ IFN-I induction

## Data Availability

No new data were created or analyzed in this study. Data sharing is not applicable to this article.

## Abbreviations

ACE2	Angiotensin-converting enzyme 2
AKI	Acute kidney injury
CD147	Cluster of Differentiation 147 (Basigin)
CDR3	Complementarity-determining region 3
COX-2	Cyclooxygenase-2
CRYO-EM	Cryo-electron microscopy
CTSL	Cathepsin L
DAMPs	Damage-associated molecular patterns
dsRNA	Double-stranded RNA
EIF	Eukaryotic initiation factor
ER	Endoplasmic reticulum
ExoN	Exoribonuclease (nsp14 domain)
FP	Fusion peptide
HLA	Human leukocyte antigen
HR1/HR2	Heptad repeat 1/2
IFN	Interferon
ISG	Interferon-stimulated gene
JAK/STAT	Janus kinase/Signal transducer and activator of transcription
KDIGO	Kidney Disease: Improving Global Outcomes
MAPK	Mitogen-activated protein kinase
MAVS	Mitochondrial antiviral signaling protein
MDA5	Melanoma differentiation-associated protein 5
MOI	Multiplicity of infection
mtROS	Mitochondrial reactive oxygen species
NRP1	Neuropilin-1
nsp	Nonstructural protein
OXPHOS	Oxidative phosphorylation
PAMPs	Pathogen-associated molecular patterns
PBMC	Peripheral blood mononuclear cell
PRRs	Pattern recognition receptors
PVNA	Pseudovirus neutralization assay
RBD	Receptor-binding domain
RdRp	RNA-dependent RNA polymerase
RIG-I	Retinoic acid-inducible gene I
RISH	RNA in situ hybridization
RTC	Replication–transcription complex
SARS-CoV-2	Severe acute respiratory syndrome coronavirus 2
SAM	S-adenosyl-L-methionine
SVALI	SARS-CoV-2–associated liver injury
TLRs	Toll-like receptors
TNF	Tumor necrosis factor
TRM	Tissue-resident memory T cell
